# Professional Reasoning in Occupational Therapy: A Scoping Review

**DOI:** 10.1155/2019/6238245

**Published:** 2019-11-26

**Authors:** Luis-Javier Márquez-Álvarez, José-Ignacio Calvo-Arenillas, Miguel-Ángel Talavera-Valverde, Pedro Moruno-Millares

**Affiliations:** ^1^Universidad de Salamanca, Salamanca 37007, Spain; ^2^Department of Nursing and Physiotherapy, Universidad de Salamanca, Escuela Universitaria de Enfermería y Fisioterapia, Salamanca 37007, Spain; ^3^Integra Saude Unit Research, Department of Health Science, Facultad de Ciencias de la Salud, Universidade da Coruña, Faculty of Health Science, A Coruña 15570, Spain; ^4^Department of Nursing, Physiotherapy and Occupational Therapy, Universidad de Castilla-La Mancha, Toledo, Talavera de la Reina 45600, Spain

## Abstract

**Background/Aim:**

Professional reasoning in occupational therapy is the process used by practitioners to plan, direct, perform, and reflect on client care. The professional's ability to manage the process of the intervention is structured around it, thereby influencing the effectiveness of the work carried out. The objectives of this research were to identify and describe (a) the historical development of this area of research from 1982 to 2017 and (b) the nature and volume of the scientific literature on professional reasoning in occupational therapy and the evidence that exists today.

**Methods:**

A scoping review method was used to carry out an historical mapping of research on professional reasoning and to summarise the lines of research explored to date. The review was conducted in five stages following the PRISMA guidelines. After applying the selection criteria, the search identified 303 references.

**Results:**

The results are presented under three headings: (a) nature and volume of publications on professional reasoning in occupational therapy according to number and year of publications, journal, country, author, and line of research; (b) historical trends in the scientific literature on professional reasoning in occupational therapy since 1982; and (c) methodological aspects of the research. Each of them is discussed through statistical analysis.

**Conclusions:**

The research about professional reasoning in occupational therapy is a field of empirical nature, in which qualitative studies predominate. Principal lines of research are focused on specific fields of practice, undergraduates, and theoretical aspects of professional reasoning. There were identified three historical phases with common features in terms of objectives and research methods.

## 1. Introduction

In occupational therapy, professional reasoning can be defined as the process used by practitioners to plan, direct, perform, and reflect on client care [1, 2]. Its importance in professional practice is fundamental given that the professional's ability to manage the process of assessing, planning, and implementing the intervention is structured around it, thereby influencing the effectiveness of the work carried out [2–4].

Currently, the scientific literature on professional reasoning in occupational therapy describes it as a highly complex mode of thought that “involves all the thinking processes of the clinician as s/he moves into, through and out of the therapeutic relationship and therapy process with a client” [4]. It is characterised as a mode of tacit, highly creative and deeply phenomenological thinking [5, 6], aimed at determining the focus of care for a given client or group of clients [1]. It is studied using a range of approaches, in terms of both focus and method [7].

Despite its importance in our discipline, the body of knowledge on professional reasoning in occupational therapy is still inadequate [8, 9]. To date, there has been no full, comprehensive review of the scientific literature that would allow us to define and summarise existing scientific evidence in the area of professional reasoning in occupational therapy. Previous reviews of the literature on clinical reasoning in occupational therapy limited the databases selected, the languages of the studies, and the analyses carried out. They were therefore subject to possible biases in the information gathered. [4, 10, 11].

For this reason, we conducted a scoping review to identify and describe the scientific publications on professional reasoning and to analyse the historical development of this area of research from 1982 to 2017 and the nature and volume of the scientific literature on professional reasoning in occupational therapy and the evidence that exists today.

## 2. Materials and Methods

A scoping review method [12–14] was used to carry out an exploratory historical mapping of research on professional reasoning and to summarise the lines of research explored to date. The review was conducted in five stages [14] following the PRISMA guidelines [15].

### 2.1. Review Question and Relevant Papers

The research questions that guided the review were as follows: (a) What is the nature and volume of the literature on professional reasoning in occupational therapy? (b) How has research on professional reasoning evolved over time? In the first stage, a two-step search strategy was employed for this review. First, an initial search strategy (January 11, 2018) was created for Medline (using Ovid) and was adapted to each search: (1) reasoning.af (16,579); (2) occupational therapy/(12,440); (3) occupational therap∗.ab,ti (10,234); (4) allied health occupations/(547); (5) allied health personnel/(11,272); (6) 2 or 3 or 4 or 5 (27,348); (7) 6 and 1 (218). In this way, we established if the terms contained in the title, abstract, or keywords of the retrieved citations allied with the planned search terms. Finally, the keywords used are classified in Table 1.

Second, the formal literature search was conducted across the selected databases: OTDBase, CINAHL, Medline, WOS, Embase, Scopus, ISOC, Latindex, LILACS, LivRe, ProQuest, CSIC (Spanish National Research Council), and Dialnet. The results were actualized on February 15, 2019. In addition to the abovementioned databases, a search was also carried out on Google Scholar (https://scholar.google.es/) and the catalogue of the Network of Spanish University Libraries (http://rebiun.org/) in order to identify further references from magazines, books, book chapters, and theses for their possible inclusion. With this search strategy, we have tried to gather information in the most thorough way possible, without limiting the language of the documents and by incorporating databases that have not been used in previous literature reviews. Our aim was to avoid any bias that could diminish the information obtained.

### 2.2. Selection of Relevant Studies

In the second stage, we proceeded to identify and select the relevant studies. The following selection criteria were established.

- Inclusion criteria: any article, book (publications dealing with professional reasoning in all their chapters), book chapter (publications that, while appearing in a book on various subjects, specifically cover the subject in question), or doctoral thesis in which any of the keywords appear in the title, keywords list, abstract, or headings of the document. Material in any language was included
- Exclusion criteria: documents that did not contain any of the keywords were excluded. Furthermore, after removing any duplicate documents, we excluded studies that did not focus on professional reasoning in occupational therapy or in health professions that would include occupational therapists

These inclusion and exclusion criteria were refined as we gained familiarity with the literature [12].

### 2.3. Data Charting

In the third stage, carried out simultaneously with stage two, the data were extracted from each 303 references and included in a data extraction table developed by the research team. This data extraction table was developed using the programme IBM SPSS Statistics (V.25). The data extraction process was carried out by researchers L.M. and M.T. independently. It was subsequently reviewed by researchers C.A. and P.M.

### 2.4. Data Sorting and Analysis

The fourth stage consisted of sorting the data following an iterative process and using the following categories: title, author, characteristics of the publication (journal or publisher, year of publication, publication type, and language), objectives of the study, and study design (type of method, type of study, methodological design of the study, and subject of the study). Our aim was to identify parameters for analysing the literature that would enable us to carry out a detailed critical review. The fifth stage involved a comprehensive review of the selected documents. After reading and analysing the articles published in indexed journal, the historical research trends since the publication of the first article in 1982 were identified [16]. Lastly, a descriptive and inferential statistical analysis was performed by applying the chi-square test to the different categories of scientific articles published between 1982 and 2014. In addition, Fisher's exact test was applied to scientific articles included in the same period with a frequency below *n* = 5 to analyse the statistically significant relationships between the variables selected in cases where the chi-square test was not representative. To carry out the statistical analyses detailed above, the articles were grouped into 10-year periods in order to compare the different phases statistically. Therefore, articles published between 2015 and 2017 were not considered in these statistical analyses.

## 3. Results

**Figure 1:**
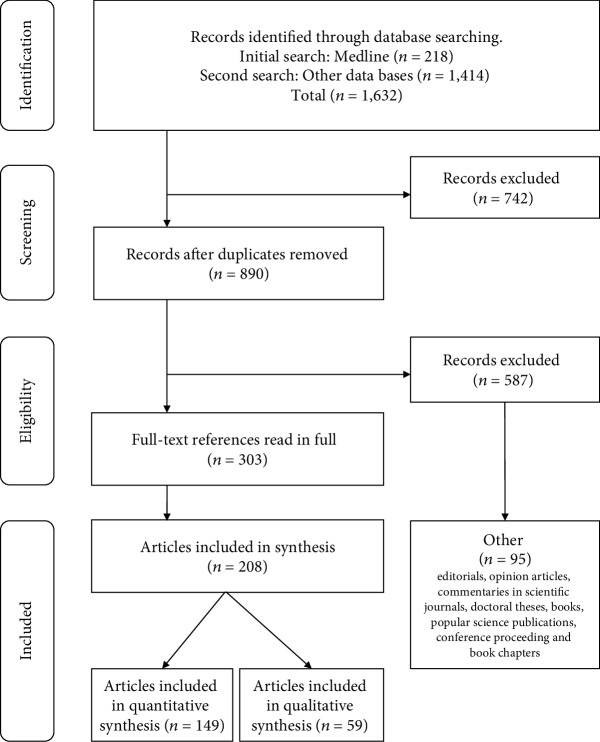
PRISMA flow diagram [15].

The search strategies retrieved 1,632 references (890 once duplicates were removed). After applying the selection criteria, we identified 303 references (Figure 1).

The results are presented under three headings: (a) nature and volume of publications on professional reasoning in occupational therapy according to number of publications, year of publication, journals, country, author, and line of research; (b) historical trends in the scientific literature on professional reasoning in occupational therapy since 1982; and (c) methodological aspects of the research.

### 3.1. Nature and Volume of Publication

Of the 303 references analysed, the largest percentage corresponds to articles published in indexed journals (original studies and reviews): *n* = 208 (68.6%). The remaining references are editorials, opinion articles, and commentaries in scientific journals, with *n* = 37 (12.2%); doctoral theses, *n* = 22 (7.3%); books, *n* = 7 (2.3%); popular science publications, *n* = 5 (1.7%); conference proceedings, *n* = 12 (4%); and book chapters, *n* = 12 (4%). With regard to the languages used by the authors, English predominates with *n* = 280 (92.4%), followed by Spanish, *n* = 14 (4.6%); German, *n* = 5 (1.7%); and French, Polish, Portuguese, and Hebrew, *n* = 1 (0.3%).

**Figure 2:**
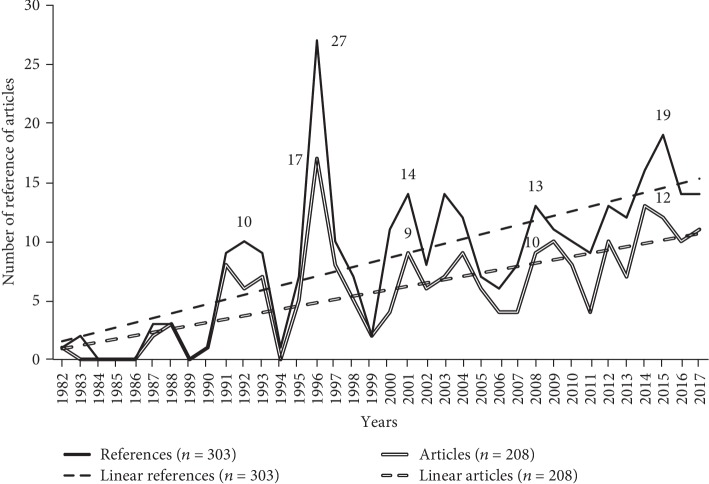
Comparison between number of documents published and articles published in indexed journals (original studies and reviews) 1982-2017.

Since 1982, a gradual and steady increase can be observed in the number of documents published (Figure 2).

The analysis reveals that the articles published in indexed journals (original studies and reviews) were published in 49 different journals, with publications in English predominating (*n* = 195; 93.8%). The journals with the largest number of articles are *the American Journal of Occupational Therapy*, with *n* = 42 (20.2%), and *the British Journal of Occupational Therapy*, with *n* = 32 (15.4%). These are followed by the *Australian Journal of Occupational Therapy*, with *n* = 18 (8.7%); *Occupational Therapy in Health Care*, with *n* = 17 (8.2%); the *Scandinavian Journal of Occupational Therapy*, with *n* = 15 (7.2%); and the *Canadian Journal of Occupational Therapy*, with *n* = 10 (4.8%). The rest of the journals fell short of 10 articles published. With regard to non-English-language journals, the greatest number of publications was found in the Spanish-language TOG (A Coruña), with *n* = 4 (1.9%).

With regard to the 439 authors, English-speaking authors overshadow the rest with *n* = 414 (94.3%). No author, except for C.A. Unsworth, with nine empirical articles and one nonempirical article, reaches a total of 10. This author is followed by Neistadt, with seven empirical articles, and Rodger and Ziviani, with five empirical articles. With regard to non-English-speaking authors, only two appear among the top 29: Talavera, with four empirical articles, and Moruno, with two.

In addition, four major lines of research were identified in the analysis of the articles published in indexed journal (original studies and reviews) (Table 2).

With regard to the books and book chapters published from 1982 to the present, an irregular pattern can be observed when compared to the scientific articles published in indexed journals. Books (57.1%) and book chapters (50%) of a theoretical nature predominate. Since 1995, the year in which the first doctoral thesis on professional reasoning in occupational therapy was published, there has been a gradual increase in the publication of doctoral theses similar to the increase observed in articles published in indexed journals. With regard to the methodology of the doctoral theses, in contrast to the articles published in indexed journals, quantitative studies predominate (57.1%; *n* = 13), followed by qualitative studies (38.1%; *n* = 8) and mixed studies (4.8%; *n* = 1). The main lines of research among the doctoral theses are student reasoning (*n* = 8; 38.1%), specific professional fields (*n* = 3; 13.6%), and novice/expert reasoning (*n* = 3; 13.6%).

### 3.2. Historical Trends

The first article focused on the study of clinical reasoning was published in 1982 [16] and aimed to define this area of study within the field of occupational therapy. The first review of the literature on clinical reasoning in occupational therapy was published in 1993 [17].

On the basis of the analysis of the articles published in indexed journals (original studies and reviews) published between 1982 and 2017 (*n* = 208), it was identified that *n* = 149 (71.6%) are empirical studies and *n* = 59 (28.4%) do not have an empirical basis. It should be noted that between 1982 and 1993, there are a similar number of nonempirical articles *n* = 10 (4.8%) and empirical ones *n* = 11 (5.3%). In that period, the articles are mainly exploratory and descriptive (Table 3).

In more recent periods, an increase can be observed in the publication of both empirical and nonempirical articles, and in the variety of methodological approaches used in the studies. The majority of the explanatory studies (*n* = 4) converge in the period 2004-2014, as does a large share of the empirical scientific output *n* = 67 (32.2%).

**Figure 3:**
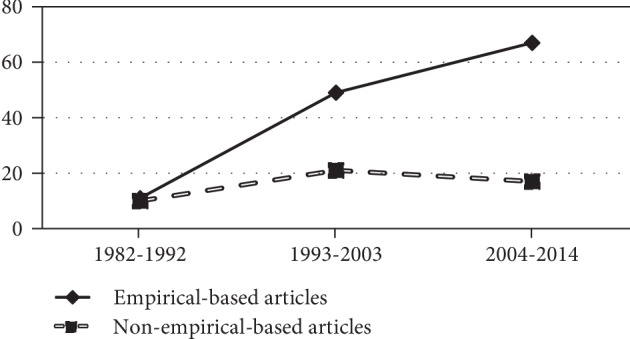
Evolution of the number of articles published in indexed journals (original studies and reviews) according to method.

Figure 3 shows an increase in both trends. The empirical trend is more dominant in recent years. By calculating their linear average, we can observe how the gap widens between the two trends, with the nonempirical trend making more limited progress.

When comparing the first three periods, which last the same amount of time (*n* = 175), a statistically significant relationship (*p* < 0.05) is found between the periods and the methodology used in the articles. There is a statistically significant relationship between nonempirical articles and the period 1982-1992 (*p* < 0.05), when compared with the other periods. Furthermore, there is a statistically significant relationship between empirical articles and the period 2004-2014 (*p* < 0.05), when compared with previous periods.

### 3.3. Methodological Aspects of the Research

The descriptive analysis of the methods used in the empirical articles is summarised in Table 4.

Overall, the percentage of qualitative articles published *n* = 72 (48.3%) exceeds the percentage of quantitative articles, mixed articles, and reviews.

During the years 1982 to 1992, we can identify a greater number of qualitative studies (*n* = 8) based on ethnographic and phenomenological approaches in comparison to quantitative and mixed studies (*n* = 3). In the case of articles using quantitative methodology, we find the same number of experimental studies and observational studies. In this period, a study categorised as “qualitative and experimental” was identified, which from our point of view is a clear methodological error, because the description provided (qualitative and experimental) does not reflect the methodology used.

During the years 1993 to 2003, an increase is observed in both quantitative studies and in reviews and mixed research designs (*n* = 17). Nevertheless, a greater number of qualitative articles (*n* = 32) continue to be published, particularly ones using a phenomenological approach (*n* = 13).

However, during the years 2004 to 2014, the trend from the previous period reverses. Quantitative studies (*n* = 29) outweigh qualitative ones (*n* = 23), and the number of literature reviews increases significantly.

In terms of possible correlations, we performed Fisher's exact test (due to the existence of values *n* < 5 in some categories) to analyse the major design approaches (quantitative, qualitative, mixed, and review) in relation to the first three periods described (*n* = 127). We can confirm that there is a significant relationship between qualitative methodology and publications during the years 1993 to 2003 (*p* < 0.01) and between quantitative methodology and publications with respect to the period 2004-2014 (*p* < 0.05).

## 4. Discussion

The results obtained in this scoping review allow us to answer the research questions posed at the outset of this paper. Regarding the first question, we have been able to describe the nature and volume of the research carried out on professional reasoning in occupational therapy. Since 1982, there has been a gradual and steady increase in the number of research articles on professional reasoning in occupational therapy, which may indicate a growing interest in this area of knowledge. In relation to this fact, it is fair to say that professional reasoning in occupational therapy has become a consolidated and ongoing line of research during the period studied.

Overall, research on professional reasoning in occupational therapy is empirical. Furthermore, qualitative research predominates, with the number of qualitative articles published exceeding the number of quantitative and mixed methodology articles and reviews. This dominance of qualitative research on this topic is likely because qualitative techniques are appropriate to the nature of research questions about clinical reasoning because they allow in-depth responses and field notes on observations of clinical reasoning in practice. In addition, it may be also related to the predominance of qualitative research in our discipline during the eighties and the nineties. There has been only one systematic review with methodological rigour, conducted by Unsworth and Baker [4]. However, it did not involve a detailed analysis of the scientific rigour of the studies.

By mapping the research topics associated with professional reasoning, we have been able to identify three major lines of study: (a) professional reasoning in specific fields of practice, (b) professional reasoning among undergraduates, and (c) theoretical aspects of professional reasoning. Other relevant lines of study include modalities of reasoning and the differences in professional reasoning between novices and experts.

In light of these results, it appears that research on professional reasoning in occupational therapy is especially concerned with the particularities of reasoning in specific professional fields, to the detriment of the study of information processing that takes place in practice and that shapes professional reasoning in general [18]. This fact is reflected in the 25 articles classified under this category (information processing). We agree with Schell et al. [19] when they suggest that research on information processing could: “…help the occupational therapy community understand the applicability and limitation of information-processing models that are borrowed from research in other professions.” (p. 410). Furthermore, there is a lack of studies focused on the distinctive and unique modalities of reasoning that occur among occupational therapists [19]. In this scoping review, only 18 papers were identified in which the different modalities of professional reasoning were the focus of research. Despite the fact that these modalities of reasoning are frequently referred to in scientific literature [17], it appears that in-depth study of procedural, interactive, conditional, ethical, and pragmatic reasoning has not yet occurred. Therefore, we call on occupational therapists to continue to move beyond the limits established by information-processing models taken from other professions and to explore in more depth the unique and distinctive characteristics of professional reasoning in occupational therapy.

It should also be noted that publications from English-speaking countries predominate, particularly the United States, Britain, Australia, and Canada, followed by publications in Northern European and Spanish-speaking countries. This suggests that the clinical reasoning of OTs in developing countries has not been sufficiently studied, which is likely to limit the progression of OT practice in these countries [20–22]. This scoping review has broadened the search criteria of previous literature reviews to try to correct this bias.

With regard to the second research question, we have been able to describe how research on professional reasoning in occupational therapy has evolved. Our findings point to three historical periods with distinct characteristics: (a) exploratory phase (1982-1993), (b) transition phase (1994-2003), and (c) consolidation phase (2005-present).

In the exploratory phase (1982-1993), the scope of the research that would be developed in later literature is defined, described, and explored. This phase is characterised by nonempirical qualitative studies based on ethnographic and phenomenological approaches, which seems to indicate an exploratory perspective [23]. This thesis is consistent with the findings of Unsworth and Baker [4] and Harries and Harries [24], and with the statistically significant relationship we have identified between the nonempirical articles published and the period 1982-1993, when compared with the other periods.

In the transition phase (1994-2003), the number of studies increases considerably, the types of studies carried out diversify and there is also a significant increase in empirical studies, which outweigh nonempirical studies during these years. This increase in empirical studies is probably related to the need to support occupational therapy with more rigorous scientific research. However, among the empirical articles published during this period, qualitative articles with a phenomenological approach predominate. According to the data analysed, this theory is consistent with the statistically significant relationship found between this phase and the use of qualitative methodology. It is likely that, although researchers were still seeking to develop a rich descriptive image of professional reasoning, the available scientific methods at that time were becoming more rigorous in the field of health sciences. These findings seem to indicate a transition period in the research, during which new research perspectives are developed, while the earlier ones continued to predominate [24].

In the consolidation phase (2005-present), the research trend is clearly reversed, with a quantitative approach predominating and an increase in the number of literature reviews. These findings indicate that, in recent decades, research on professional reasoning has reached a period of consolidation, adopting a variety of both qualitative and quantitative approaches, although qualitative studies still predominate [25]. This thesis is consistent with the statistically significant relationship found here regarding empirical articles using quantitative methodology and the period 2004-2014, when compared with previous years. In addition, almost a third of the studies published during that period were reviews and experimental designs, which indicates a research trend to achieve a higher level of scientific evidence.

### 4.1. Limitations

A detailed analysis of the findings of the papers included in this review was beyond the scope of this study. Moreover, this scoping review did not assess the scientific quality of the literature analysed, which may be considered a limitation of the study.

### 4.2. Future Research

Future lines of research need to assess the methodological quality and scientific evidence arising from studies on professional reasoning in occupational therapy. From our point of view, conducting a study to assess the quality of the publications and the existing evidence is imperative.

It would be interesting for research in this area to encompass a greater number of non-English-speaking countries in order to gather information about the cultural and ethical particularities of professional reasoning [8, 19, 26].

## 5. Conclusions

Research and literature about professional reasoning in occupational therapy is a rising field of knowledge, through which occupational therapists increase their understanding of the mechanisms that regulate the selection and evaluation of occupational therapy interventions. The research about professional reasoning in occupational therapy has increasingly involved empirical research, in which qualitative studies predominate. However, there is still a relative lack of quantitative and mixed methods studies, as well as a dearth of systematic reviews about the quality of existing studies. Principal lines of research focus on specific fields of practice, undergraduates, and theoretical aspects of professional reasoning. There are relatively few studies focused on information processing, modalities, and unique characteristics of professional reasoning in occupational therapy. Three historical phases were identified with common features in terms of objectives and research methods: (a) exploratory phase, characterised by nonempirical studies; (b) transition phase, in which there is a considerable increasing diversification of the lines and methods of research; and (c) consolidation phase, in which evidence-based research perspectives and more quantitative studies emerge. Overall, the research about professional reasoning in occupational therapy during the next years should target the in-depth study of the basic process of information processing and the reasoning modalities that define the occupational therapy professional reasoning.

## Figures and Tables

**Table 1:** List of descriptors and keywords used in the search.

MeSH	DeCS	Keywords
Occupational therapy	Terapia Ocupacional	Reasoning
Allied health occupations	Empleos Relacionados con Salud	Allied health personnel
Problem solving	Solución de Problemas	Clinical reasoning
Patient care planning	Planificación de Atención al Paciente	Professional reasoning
Decision-making	Toma de Decisiones	
Cognition	Cognición	

**Table 2:** Number of articles published in indexed journal (original studies and reviews) published between 1982 and 2017 by line of research.

	1982-1992 (*n* = 21)	1993-2003 (*n* = 70)	2004-2014 (*n* = 84)	2015-2017 (*n* = 33)	Total (*n* = 208)
	*n* (%)	*n* (%)	*n* (%)	*n* (%)	*n* (%)
Theoretical	3 (1.4)	11 (5.3)	12 (5.8)	9 (4.3)	35 (16.8)
Student reasoning	4 (1.9)	13 (6.3)	19 (96.1)	6 (2.9)	42 (20.2)
Information processing	3 (1.4)	12 (5.8)	5 (2.4)	5 (2.4)	25 (12)
Specific professional fields					
The elderly	1 (0.5)	2 (1)	2 (1)	0 (0)	5 (2.4)
Mental health/psychosocial	2 (1)	1 (0.5)	4 (1.9)	1 (0.5)	8 (3.8)
Schools	1 (0.5)	0 (0)	0 (0)	0 (0)	1 (0.5)
Spinal cord injury	0 (0)	1 (0.5)	0 (0)	0 (0)	1 (0.5)
Cancer	0 (0)	1 (0.5)	1 (0.5)	0 (0)	2 (1)
Neurology	0 (0)	1 (0.5)	6 (2.9)	1 (0.5)	8 (3.8)
Hand damage	0 (0)	0 (0)	2 (1)	0 (0)	2 (1)
Community	0 (0)	4 (1.9)	3 (14)	0 (0)	7 (3.4)
Support/accessibility technology	0 (0)	0 (0)	1 (0.5)	4 (1.9)	5 (2.4)
Paediatrics	0 (0)	2 (1)	6 (2.9)	2 (1)	10 (4.8)
Physical disability	1 (0.5)	3 (1.4)	4 (1.9)	1 (0.5)	9 (4.3)
Other lines					
Novice/expert	1 (0.5)	7 (3.4)	5 (2.4)	3 (1.4)	16 (7.7)
Modalities of reasoning	5 (2.4)	7 (3.4)	6 (2.9)	0 (0)	18 (8.7)
Assistants	0 (0)	2 (1)	0 (0)	0 (0)	2 (1)
Research methodology	0 (0)	3 (1.4)	5 (2.4)	1 (0.5)	9 (4.3)
Cultural aspects and contexts	0 (0)	0 (0)	3 (1.4)	0 (0)	3 (1.4)

The percentages were calculated on the basis of the sample of articles published in indexed journals (original studies and reviews) (*n* = 208).

**Table 3:** Number of articles published in indexed journal (original studies and reviews) published between 1982 and 2017 by study type.

	1982-1992 (*n* = 21)	1993-2003 (*n* = 70)	2004-2014 (*n* = 84)	2015-2017 (*n* = 33)	Total (*n* = 208)
	*n* (%)	*n* (%)	*n* (%)	*n* (%)	*n* (%)
Empirical articles					
Exploratory	5 (2.4)	16 (7.7)	24 (11.5)	8 (3.8)	53 (25.5)
Descriptive	4 (1.9)	19 (9.1)	20 (9)	7 (3.4)	50 (24)
Correlation	1 (0.5)	4 (1.9)	11 (5.3)	3 (1.4)	19 (9.1)
Scoping	1 (0.5)	9 (4.3)	8 (3.8)	4 (1.9)	22 (10.6)
Explanatory	0 (0)	1 (0.5)	4 (1.9)	0 (0)	5 (2.4)
Non-empirical articles	10 (4.8)	21 (10.1)	17 (8.2)	11 (5.3)	59 (28.4)

The percentages were calculated on the basis of the sample of articles published in indexed journals (original studies and reviews) (*n* = 208).

**Table 4:** Number of articles published in indexed journals (original studies and reviews) using empirical methods published between 1982 and 2017 by study design.

	1982-1992 (*n* = 11)	1993-2003 (*n* = 49)	2004-2014 (*n* = 67)	2015-2017 (*n* = 22)	Total (*n* = 149)
	*n* (%)	*n* (%)	*n* (%)	*n* (%)	*n* (%)
Quantitative					
Experimental	1 (0.7)	3 (2)	13 (8.7)	4 (2.7)	21 (14.1)
Cross-sectional non-experimental	0 (0)	7 (4.7)	11 (7.4)	3 (2)	21 (14.1)
Longitudinal nonexperimental	1 (0.7)	2 (1.3)	5 (3.4)	0 (0)	8 (5.4)
Qualitative					
Experimental	1 (0.7)	0 (0)	0 (0)	0 (0)	1 (0.7)
Cross-sectional nonexperimental	0 (0)	1 (0.7)	0 (0)	0 (0)	1 (0.7)
Grounded theory	0 (0)	0 (0)	4 (2.7)	4 (2.7)	8 (5.4)
Ethnographic design	3 (2)	8 (5.4)	6 (4)	3 (2)	20 (13.6)
Phenomenological design	3 (2)	13 (8.7)	9 (6)	1 (0.7)	26 (17.7)
Action-research design	1 (0.7)	1 (0.7)	1 (0.7)	1 (0.7)	4 (2.7)
Narrative design	0 (0)	9 (6)	3 (2)	0 (0)	12 (8.2)
Mixed					
Concurrent	1 (0.7)	1 (0.7)	3 (2)	1 (0.7)	6 (4.0)
Sequential	0 (0)	1 (0.7)	4 (2.7)	1 (0.7)	6 (4.0)
Integrated	0 (0)	0 (0)	0 (0)	1 (0.7)	1 (0.7)
Review	0 (0)	3 (2)	8 (5.4)	3 (2)	14 (9.4)

The percentages were calculated on the basis of the sample of empirical articles (*n* = 149).
